# Combined Low‐Level Laser and Ultrasound Therapy for Growing Pains in Children Aged Four to Twelve Years: A Pilot Study

**DOI:** 10.1002/jbio.70275

**Published:** 2026-04-29

**Authors:** Esther Angelica Luiz Ferreira, Lucas Pereira Liberal, Antonio Eduardo de Aquino Junior, Rodrigo Bezerra de Menezes Reiff, Fernanda Mansano Carbinatto, Ricardo Patrezi Zanatta, Matheus Henrique Camargo Antonio, Noahn Gabriel Silva Pereira, Maycon Rodrigo Sarracini, Vanderlei Salvador Bagnato

**Affiliations:** ^1^ Federal University of São Carlos São Carlos São Paulo Brazil; ^2^ Institute of Physics of São Carlos University of São Paulo São Carlos São Paulo Brazil; ^3^ Department of Biomedical Engineering Texas A&M University College Station Texas USA

**Keywords:** child, laser therapy, musculoskeletal pain, ultrasonic therapy

## Abstract

Growing pains are a frequent form of musculoskeletal pain in children, with an uncertain etiology and currently limited therapeutic options. Considering the need for non‐pharmacological approaches, the potential of low‐level laser therapy combined with ultrasonic therapy stands out as a possible therapeutic technique for this condition. The objective of this study was to analyze the safety and efficacy of this treatment in children diagnosed with growing pains in order to justify the development of further research on the same topic. After confirmation of the diagnosis, participants were randomized into a treatment group, treated with the laser and ultrasound‐emitting equipment, and a placebo group. Pain was assessed at three time points and recorded in a diary for 30 days. The results indicated clinical safety and possible efficacy, reinforcing the need for further clinical trials on the subject.

## Graphical abstract

This pilot study evaluated low‐level laser therapy combined with ultrasonic therapy in children with growing pains. Results demonstrated clinical safety and potential efficacy of this non‐pharmacological approach, supporting the need for further randomized clinical trials. Summary of the methodology, quantitative and qualitative analysis, and results of the pilot study conducted.

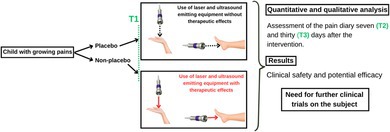

## 1 Introduction

Chronic pain is defined as persistent or recurrent pain lasting for 3 months or more. This condition affects approximately 20.8% of the pediatric population and is associated with a significant impairment of quality of life, which may even extend into adulthood [1]. Despite its high prevalence and recognition as a global public health problem, the clinical management of this condition remains largely neglected in health systems, especially due to a shortage of professionals trained in pediatric pain and a lack of knowledge about effective therapies with an adequate safety profile [2]. One of the most common causes of chronic musculoskeletal pain in children is growing pains, with a prevalence varying between 2.6% and 36.9%, depending on the study and the population analyzed [3].

Growing pains typically begin between the ages of three and twelve, characterized by painful episodes that occur mainly at the end of the day or during the night and may even interrupt sleep [4]. They present an intermittent pattern, frequently alternating between days with pain and asymptomatic days, and spontaneously cease in the morning. The clinical picture consists of bilateral, cramping pain, typically distributed in the anterior thigh, calf, and posterior knee muscles, typically without limitation of movement. Despite the typical clinical presentation, diagnostic criteria for growing pains have not yet been established. The diagnosis is made by exclusion since the patient's physical examination is normal and there is no evidence of underlying traumatic or inflammatory processes [5].

Growing pains are known to be self‐limiting, with most symptoms resolving spontaneously by adolescence [6]. Although multiple hypotheses have been proposed, their etiology and pathophysiology remain undetermined, which imposes significant limitations on the development of effective therapeutic strategies [7]. There is a significant gap in the literature regarding specific, evidence‐based interventions for managing this condition. Because of this, current therapies are mainly limited to the use of anti‐inflammatory drugs, stretching of the lower limbs, and massage of the painful areas—measures that, however, have not proved effective for all children [8]. Given this scenario, there is a search for therapies specifically targeted at growing pains that encompass the well‐being and safety of the pediatric population, with an emphasis on the use of non‐pharmacological measures, especially low‐level laser therapy combined with ultrasound therapy [9].

It is believed that low‐level laser therapy, through enzymatic modulation within the mitochondria, promotes increased ATP production when in contact with cells, leading to protein and cell proliferation and the sensitization of neurons by inflammatory mediators [10]. This technology has been used for medical treatment since the 1980s, with evidence demonstrating benefits in tissue repair, reduction of inflammatory and edematous processes, and pain relief, standing out for its non‐invasive and non‐thermal nature [11].

Ultrasound therapy, in turn, is based on the emission of high‐frequency vibrations that, upon contact with body tissues, increase cellular permeability, reduce the speed of nerve impulse conduction, and promote systemic vasodilation. It is a widely used intervention in the management and reduction of muscle pain [12]. The combination of such therapies has already been explored in the treatment of various musculoskeletal conditions, reflecting significant potential for the management of chronic pain in previous studies [13, 14].

Although low‐level laser therapy is widely used in adults, its application in children requires special attention due to the presence of epiphyseal plates, sensitive structures that, if damaged, can cause growth alterations. Studies involving animal models have shown that wavelengths in the red spectrum (620–700 nm) penetrate superficially into body tissues, limited to the skin and muscle layers, and are attenuated through the thickness of the tissues. These characteristics substantially reduce the energy that can reach deep bone structures, such as the epiphyses [15]. Furthermore, a review of studies on the effect of low‐level laser on the epiphyseal growth plate showed that, in most studies conducted in rodents, no significant differences in bone growth were observed between the treated and control groups, with no evidence of epiphyseal damage [16]. This study opted to apply the equipment to the palms of the hands and soles of the feet—regions distant from the epiphyses of the long bones—and established an explicit safety area that excluded the digital extremities, where the distal epiphyses of the fingers are located. This methodological choice aims to minimize any potential risk to the growth structures, even with the use of a surface‐penetrating wavelength.

Regarding ultrasound therapy, studies in rabbits suggest that the application of 0.5 W/cm^2^ does not induce histological changes in the growth plate, making it safe in an experimental context [17]. Furthermore, there are no reports of growth disorders in humans related to the use of ultrasound at safe doses; however, current literature emphasizes caution when applying ultrasound to regions close to the epiphyses [18]. In the present study, an intensity of 0.5 W/cm^2^ in pulsed mode was selected based on this safety threshold, and the application was performed in palmoplantar regions anatomically distant from the epiphyses of the long bones.

Considering the high prevalence of growing pains in childhood, their negative impact on the quality of life of children and their families, as well as the limited number of studies on the safety and effectiveness of non‐pharmacological therapies for managing this condition, this pilot study proposes a potentially promising therapeutic approach, aiming to broaden the spectrum of available and evidence‐based treatments for growing pains. This pilot study corresponds to the first stage of a project that aims to conduct larger‐scale randomized clinical trials involving this therapeutic modality in the pediatric population. The objective of the study was to evaluate the safety of low‐level laser therapy combined with ultrasound therapy in children aged four to 12 years, as well as to investigate whether this intervention exerted analgesic effects in children in this age group diagnosed with growing pains.

## 2 Materials and Methods

This was a pilot observational case–control study with a mixed‐methods analysis, developed by the Pain and Palliative Care Studies Center of the Federal University of São Carlos in partnership with the São Carlos Institute of Physics at the Santa Casa de Misericórdia of São Carlos. After approval by the Ethics Committee, data collection took place between September 2024 and August 2025. Children aged four to 12 years with typical signs and symptoms of growing pains were recruited for the research through an online form. Those who, after evaluation by an orthopedic physician from the research group, did not meet the diagnostic criteria for growing pains, as well as children with a history of hypersensitivity to light or laser therapy, and/or those whose guardians did not sign the Informed Consent Form, were excluded from the study.

Initially, the participants and their caregivers were assessed by members of the research group. At that time, the following factors were verified for each child: (1) the characteristics of the pain; (2) the intensity of the pain, using the Visual Analogue Scale (VAS), a validated and widely used tool for the pediatric age group [19] (3) the time of day when the pain was felt; (4) the factors that improved or worsened the pain; (5) the therapeutic measures previously used to control the pain; (6) the frequency of the pain (for example: how many times did your child feel this type of pain in the last week and in the last month?); (7) how the pain interfered with daily activities (play, sleep, school performance, social relationships, family relationships, etc.). In addition, a physical examination of the child was performed by a physician participating in the research group.

Subsequently, the children were divided into two groups: placebo and non‐placebo through a blind draw conducted by a physiotherapist who was responsible for applying the laser‐ and ultrasound‐emitting equipment to all participants. Neither the other members of the research team, nor the guardians, nor the children themselves knew which group each participant had been assigned to, thus ensuring the double‐blind nature of the intervention. This division aimed to compare the safety and effectiveness of the device as a potential therapeutic strategy for managing pediatric pain between the two experimental groups.

A single session was conducted using equipment that combines low‐intensity laser therapy with ultrasound therapy, allowing for synergy between the two modalities. The palms of the hands and soles of the feet were chosen as the intervention areas due to their high vascularization and innervation, which favor the absorption and systemic distribution of the photobiomodulatory effects of the therapy.

**FIGURE 1:**
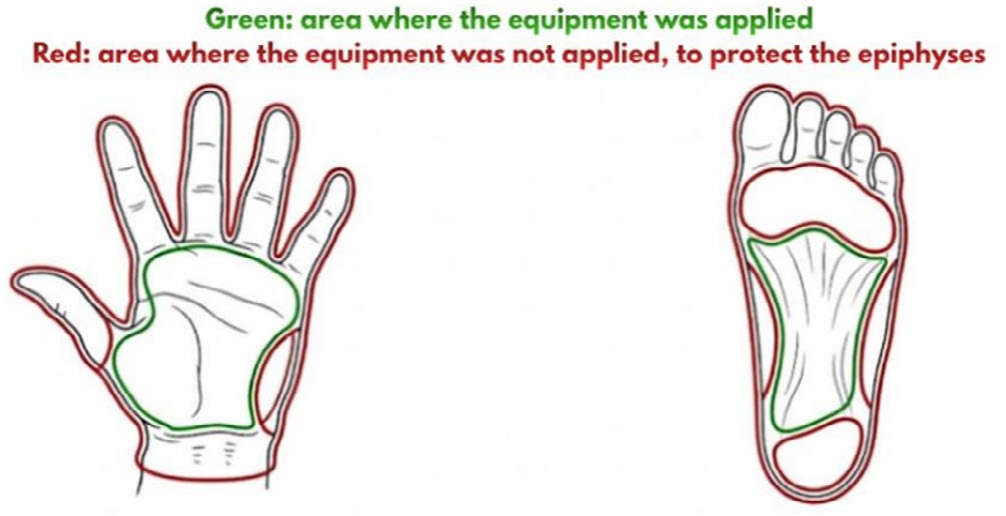
Application points for laser and ultrasound emitting equipment.

Prior to the intervention, a safety area was marked on each participant, excluding the digits, where the distal epiphyses of the phalanges are located. Consequently, the application was restricted to the central palmar and plantar regions, delimited by the distal third of the wrist and the middle third of the soles (Figure 1). The equipment was kept in contact with the skin using slow and continuous movements, traversing the previously delimited area for 3 min in each region, totaling 6 min of active application per participant. The equipment usage parameters were:

– Laser: Red wavelength (660 nm). Continuous emission.
– Ultrasound: 1 MHz, pulsed mode, secondary pulse Sata 48 Hz, intensity 0.5w/cm^2^, power 1.30 W.
– Application time: Three minutes on the palms of the hands and soles of the feet.
– Mark the safety area on the hands and the corresponding area on the feet. The finger area was disregarded [14, 20, 21].

The same device was applied to the placebo group, also in a single intervention, but without the activation of the laser and ultrasound, so that, for the participants, caregivers, and researchers, the visual process was the same; however, it was without the therapeutic effects. The children and caregivers were instructed to complete a pain diary daily for 30 days, so that the effects of laser therapy combined with ultrasound therapy could be evaluated.

The diary was evaluated by the research team at seven (T2) and thirty (T3) days after the intervention, analyzing the following factors on the days the diary was completed: (1) the self‐perception of sleep quality; (2) the daily routine and habits; (3) the pain intensity throughout the day and at the time of completing the diary (assessed using the VAS); (4) the location of the pain throughout the day and at the time of completing the diary; (5) the feelings of the day (irritability, loneliness, sadness, social relationships, etc.); (6) a free report (a section where parents or children could freely write about their behavior, feelings, and impressions of the day). The physical examination was repeated at the diary analysis points. These evaluations allowed for a comparison of participants before and after receiving the intervention, as well as a comparison of the response of the placebo and non‐placebo groups to it.

The sample size for this study was defined in accordance with the objectives of a pilot study, whose central purpose was to evaluate the feasibility and parameters necessary for larger‐scale investigations on the subject. Furthermore, by integrating quantitative and qualitative analyses, researchers were able to broaden the depth and variety of the data obtained, favoring a more comprehensive understanding of the phenomenon studied. Thus, even with a small sample, this study presented significant relevance, especially regarding the preliminary interpretation of the findings and the definition of methodological guidelines for future clinical trials involving the combined use of low‐level laser therapy and ultrasound therapy in the treatment of growing pains [22].

This was a mixed‐methods study in which a quantitative analysis of the mean reduction in the VAS (Visual Analogue Scale) before and after the intervention was performed in the placebo and non‐placebo groups. Values were collected before (T1), 7 days after (T2), and 30 days after (T3) the use of the equipment. Based on this, the mean reduction was calculated by comparing the VAS measurements at T1 with those at T2 and T3, respectively, using the Stata 13.0 software [23]. To compare the reduction in scores between groups, the Mann–Whitney U test was used. For intragroup comparisons, the Wilcoxon test was applied. The significance level adopted was $*p* < 0.05$. The qualitative analysis of the pain diaries aimed to verify the presence of side effects after the intervention, such as impairment of daily habits, disturbed sleep, increased pain, and alterations in the growth and physical examinations of the participants. In addition, the free reports provided by the caregivers were considered.

The design and reporting followed the recommendations of the STROBE Statement for case–control studies [24]. The research was approved by the Ethics Committee (CAAE 84134024.0.0000.8148). All guardians signed the Informed Consent Form and, where applicable, the children provided their assent.

## 3 Results

Seventeen children potentially eligible for the study were recruited, of whom nine were included in the research (five boys and four girls), with five in the non‐placebo group and four in the placebo group. Two children were 7 years old, one was 8 years old, one was 9 years old, two were 10 years old, two were 11 years old, and one was 12 years old. The remaining eight children were excluded because, after clinical evaluation by the orthopedic physician, the diagnosis of growing pains was not confirmed, and an alternative etiology was identified.

In an individual analysis, it was observed that there was a reduction in or maintenance of the VAS values at seven (T2) and 30 days (T3) after the intervention in all participants, when compared with the VAS values obtained at T1. More details can be seen in Table 1.

**TABLE 1:** Visual analogue scale (VAS) values at the three points in which it was applied.

	VAS at T1	VAS at T2	VAS at T3
Placebo			
Participant 2	8/10	6/10	4/10
Participant 4	6/10	No new episodes of pain	No new episodes of pain
Participant 6	5/10	5/10	2/10
Participant 8	6/10	3/10	3/10
Non‐placebo			
Participant 1	9/10	6/10	5/10
Participant 3	8/10	6/10	6/10
Participant 5	10/10	7/10	3/10
Participant 7	7/10	1/10	1/10
Participant 9	9/10	7/10	7/10

Abbreviations: T1: before applying the low‐intensity laser and ultrasound emitting equipment; T2: seven days after applying the low‐intensity laser and ultrasound emitting equipment; T3: thirty days after applying the low‐intensity laser and ultrasound emitting equipment; VAS: visual analogue scale.

In both groups, there was an average reduction in pain at both time points analyzed, with the non‐placebo group showing greater reductions than the placebo group. The comparison between groups demonstrated a greater therapeutic effect of the active intervention compared with the placebo, especially in the T1–T2 interval. These findings can be seen in Table 2.

**TABLE 2:** Average reduction in visual analogue scale (VAS) and worsening of pain when comparing the values obtained at T1, T2, and T3 in the two groups.

Moment of VAS analysis	Group	Average reduction of VAS	Worsen
Seven days after the procedure (T2)	Non‐placebo (*n* = 5)	–3.2	0%
	Placebo (*n* = 4)	–2.75	0%
Thirty days after the procedure (T3)	Non‐placebo (*n* = 5)	−4.2	0%
	Placebo (*n* = 4)	−4.0	0%

Abbreviations: T1: before applying the low‐intensity laser and ultrasound emitting equipment; T2: seven days after applying the low‐intensity laser and ultrasound emitting equipment; T3: thirty days after applying the low‐intensity laser and ultrasound emitting equipment.

At the two time points at which patients were evaluated after the intervention with the laser and ultrasound device (seven and 30 days later), there were no changes with respect to the physical examinations performed previously, such as: changes in gait patterns, pain during limb mobilization, skin lesions at the site where the device was applied, or the presence of systemic adverse reactions. Regarding the analysis of the pain diaries, an improvement in the clinical condition of seven children was observed (all from the non‐placebo group and half of the placebo group). Among the factors cited by the caregivers or the children, the following stood out: a reduction in the frequency of the children's pain complaints, in the use of usual therapeutic measures for growing pains and night awakenings, as well as a greater willingness on the part of the children to perform daily habits. No child or caregiver reported a worsening of the clinical condition after the intervention.

## 4 Discussion

The main finding of this study was the clinical safety of using low‐level laser therapy combined with ultrasound therapy to treat growing pains in children aged four to 12 years, as no adverse effects were observed in any of the children participating in the research due to the use of the laser and ultrasound equipment. Furthermore, through the analysis of the VAS (Visual Analogue Scale) scores at three different time points and by comparing the two groups, greater analgesia was observed in the non‐placebo group, indicating possible therapeutic effects of this treatment modality in children diagnosed with growing pains.

When testing a new therapeutic approach, especially in the pediatric population, clinical safety is a fundamental factor, as it ensures the well‐being of the child and their family and protects the population against avoidable harm. Unfortunately, there is currently a scarcity of research and studies addressing the safety of therapeutic interventions aimed at managing chronic pediatric pain, both pharmacological and non‐pharmacological. In the present study, the safety assessment is of great importance, especially when testing a new treatment modality for growing pains—a clinical condition for which analgesic measures are still limited—in order to ensure effective care and the well‐being of the participants throughout the research. Thus, this finding provides support for future research on the same topic [25, 26].

The development of this research helps overcome current barriers to the management of pediatric pain [27]. The use of a pain diary promoted a longitudinal and individualized assessment of the pain experience, with the active involvement of the child and their caregivers in a process that is extremely important for its proper management: the recognition of pain [28]. Through daily reports, caregivers were able to understand how the children felt throughout the study, draw conclusions about the treatment, and share them with the researchers during reassessment sessions. This contact optimized the evaluation of the pain experienced by the children after the use of low‐level laser therapy combined with ultrasound therapy.

The VAS (Visual Analogue Scale) is a validated tool for self‐reporting pain in the pediatric population, mainly due to the difficulty children have in verbally expressing what they feel [29]. Being a subjective measure susceptible to individual variations, the VAS corroborates the multifactorial nature of pain and the episodic course of growing pains. Such factors can lead to a reduction in pain intensity even in the absence of a specific therapeutic effect [30]. In this context, the use of a placebo group was important so that the improvement would not be attributed solely to the group that received the intervention, broadening the scope of the analysis of the effects of the laser‐ and ultrasound‐emitting equipment in children with growing pains.

This study has limitations. The small sample size may not be sufficient for robust and generalizable statistical analyses. However, the preliminary results indicate that the combination of low‐level laser therapy with ultrasound therapy demonstrates promise in managing growing pains. The observation of a more significant average reduction in the VAS scores in the active intervention group after the application of the combined therapy suggests the possible efficacy of the intervention. Recent pilot studies involving the pediatric population indicate that the favorable initial results of innovative therapies for pain management justify continued investigation, with the aim of conducting larger‐scale controlled clinical trials to allow for a more precise evaluation of the applicability and effectiveness of these approaches [31, 32].

## 5 Conclusion

Low‐level laser therapy combined with ultrasound therapy is safe and potentially effective for treating growing pains in children aged four to 12 years. In a context where pediatric pain is neglected and treatments for growing pains are limited, the development of new, safe therapies is of utmost importance to promote the well‐being of children and their caregivers.

The clinical safety demonstrated by the use of the laser and ultrasound equipment in the children who participated in this pilot study provides a solid basis for the development of subsequent clinical trials with larger sample sizes and longer follow‐up periods in order to verify the effectiveness of this combined therapeutic modality for the treatment of growing pains.

## Funding

This work was supported by Conselho Nacional de Desenvolvimento Científico e Tecnológico.

## Ethics Statement

This study was approved by the research ethics committee of the participating institution: CAAE 84134024.0.0000.8148.

## Consent

The guardians of all children who participated in the research signed an Informed Consent Form, and children older than 8 years signed an Informed Assent Form.

## Conflicts of Interest

The authors declare no conflicts of interest.

## Data Availability

The data that support the findings of this study are available from the corresponding author upon reasonable request.
